# Identification of novel and potent inhibitors of SARS-CoV-2 main protease from DNA-encoded chemical libraries

**DOI:** 10.1128/aac.00909-24

**Published:** 2024-08-28

**Authors:** Dario Akaberi, Monireh Pourghasemi Lati, Janina Krambrich, Julia Berger, Grace Neilsen, Emilia Strandback, S. Pauliina Turunen, Johan Wannberg, Hjalmar Gullberg, Martin Moche, Praveen Kumar Chinthakindi, Tomas Nyman, Stefan G. Sarafianos, Anja Sandström, Josef D. Järhult, Kristian Sandberg, Åke Lundkvist, Oscar Verho, Johan Lennerstrand

**Affiliations:** 1 Department of Medical Biochemistry and Microbiology, Zoonosis Science Center, Uppsala University, Uppsala, Sweden; 2 Department of Medicinal Chemistry, Uppsala University, Uppsala, Sweden; 3 Department of Medical Sciences, Clinical Microbiology, Uppsala University, Uppsala, Sweden; 4 Center for ViroScience and Cure, Laboratory of Biochemical Pharmacology, Department of Pediatrics, Emory University School of Medicine, Atlanta, Georgia, USA; 5 Children’s Healthcare of Atlanta, Atlanta, Georgia, USA; 6 Department of Medical Biochemistry and Biophysics, Protein Science Facility, Karolinska Institutet, Stockholm, Sweden; 7 Department of Protein Science, School of Engineering Sciences in Chemistry, Biotechnology and Health, KTH Royal Institute of Technology, Solna, Sweden; 8 Drug Discovery and Development, Science for Life Laboratory, Solna, Sweden; 9 Department of Medicinal Chemistry, Science for Life Laboratory, BMC, Uppsala University, Uppsala, Sweden; 10 Science for Life Laboratory, Biochemical and Cellular Assay Facility, Drug Discovery and Development Platform, Department of Biochemistry and Biophysics, Stockholm University, Solna, Stockholm, Sweden; 11 The Beijer Laboratory, Department of Medicinal Chemistry, Drug Design and Discovery, Uppsala University, Uppsala, Sweden; 12 Department of Medical Sciences, Zoonosis Science Center, Uppsala University, Uppsala, Sweden; 13 Science for Life Laboratory, Drug Discovery & Development Platform, Uppsala University, Uppsala, Sweden; IrsiCaixa Institut de Recerca de la Sida, Barcelona, Spain

**Keywords:** coronaviruses, SARS-CoV-2, protease inhibitors, DNA-encoded chemical library (DECL), M^pro^, antivirals

## Abstract

*In vitro* screening of large compound libraries with automated high-throughput screening is expensive and time-consuming and requires dedicated infrastructures. Conversely, the selection of DNA-encoded chemical libraries (DECLs) can be rapidly performed with routine equipment available in most laboratories. In this study, we identified novel inhibitors of SARS-CoV-2 main protease (M^pro^) through the affinity-based selection of the DELopen library (open access for academics), containing 4.2 billion compounds. The identified inhibitors were peptide-like compounds containing an N-terminal electrophilic group able to form a covalent bond with the nucleophilic Cys145 of M^pro^, as confirmed by x-ray crystallography. This DECL selection campaign enabled the discovery of the unoptimized compound SLL11 (IC_50_ = 30 nM), proving that the rapid exploration of large chemical spaces enabled by DECL technology allows for the direct identification of potent inhibitors avoiding several rounds of iterative medicinal chemistry. As demonstrated further by x-ray crystallography, SLL11 was found to adopt a highly unique U-shaped binding conformation, which allows the N-terminal electrophilic group to loop back to the S1′ subsite while the C-terminal amino acid sits in the S1 subsite. MP1, a close analog of SLL11, showed antiviral activity against SARS-CoV-2 in the low micromolar range when tested in Caco-2 and Calu-3 (EC_50_ = 2.3 µM) cell lines. As peptide-like compounds can suffer from low cell permeability and metabolic stability, the cyclization of the compounds will be explored in the future to improve their antiviral activity.

## INTRODUCTION

Four years after the COVID-19 pandemic started, infections are driven by the emergence of new SARS-CoV-2 variants of concern (1). Although mRNA vaccines have been instrumental in reducing severe disease and hospitalization, achieving long-term immunity appears challenging, and periodical boosting is required. The now dominant Omicron subvariants present over 30 mutations in the spike protein (S gene), conferring resistance to neutralizing antibodies induced by previous mRNA vaccines, bivalent vaccines, or infection with a previous variant (2–4). Also, the efficacy of monoclonal antibodies used to prevent SARS-CoV-2 infection (5), disease progression, and death (6, 7) can be reduced by emerging variants presenting new mutations in the spike gene (6, 7). In this scenario, antiviral options for the prevention and treatment of SARS-CoV-2 infection in immunosuppressed and high-risk subjects are necessary to further reduce the COVID-19 burden. Moreover, broad-spectrum antivirals active against several coronaviruses will be instrumental in preventing or mitigating the next pandemic by reducing early transmission and providing a starting point for the development of more potent compounds if necessary.

SARS-CoV-2 3-chymotrypsin-like cysteine protease (3CL protease) also known as main protease (M^pro^) (8) mediates the maturation of viral proteins by cleaving the two viral polyproteins pp1a and pp1ab at 11 sites (9). M^pro^ represents an attractive target for the development of antivirals against SARS-CoV-2 as it is essential for the viral life cycle and structurally conserved among alpha and beta coronaviruses (10), allowing the development of potent pan-coronavirus protease inhibitors (11–13). The feasibility of M^pro^ inhibitors as prophylaxis or treatment against SARS-CoV-2 infection has also already been proven. Two orally administered M^pro^ inhibitors, nirmatrelvir (approved by the FDA and EMA) and ensitrelvir (approved in Japan by the Ministry of Health, Labour and Welfare (14)), are currently approved for the emergency treatment of COVID-19.

SARS-CoV-2 inhibitors have been identified through the development of substrate-derived peptide-like compounds (8, 15, 16) as well as screening of large libraries of compounds and fragments using different techniques such as *in silico* screening (11), high-throughput screening (17), crystallographic screening (18), or a combination of these techniques (19).

Studies have also reported the identification of novel M^pro^ inhibitors, with half-maximal inhibitory concentration (IC_50_) and EC_50_ in the low micromolar range (20–22) and nanomolar range (23), using DNA-encoded chemical libraries (DECLs). DECLs are large collections of compounds tagged with a unique DNA barcode that is screened by affinity to select binders for a target of interest (24). Following the selection process, the chemical structures of the binders are elucidated through the sequencing of their unique DNA tags. This technology is particularly attractive because the combinatorial nature of DECL combined with affinity screening allows for rapid and cost-efficient exploration of large portions of chemical space *in vitro*.

Here, we report a new class of peptide-like inhibitors of M^pro^ identified using the commercially available DELopen platform (WuXi AppTec) that provides a library of 4.2 billion compounds (screened by the user) as well as the services necessary for the sequence to structure decoding of binders’ DNA tags. The most potent compound (MP1) inhibited the activity of SARS-CoV-2 M^pro^ with IC_50_ = 24 nM and SARS-CoV-2 infection with EC_50_ = 2.3 µM in cell-based assays.

## MATERIALS AND METHODS

### Bead capture test

Immobilization of M^pro^-Avi to Dynabeads MyOne Streptavidin T1 paramagnetic beads was tested before affinity selection according to a protocol provided with the DELopen kit. All reagents were added to 1.5-mL Eppendorf DNA Low Binding tubes during the bead capture test and affinity selection. In brief, paramagnetic beads were washed three times with 1× wash buffer (50 mM Tris-HCl pH 7.5–150 mM sodium chloride–0.05% Tween-20–1 mM dithiothreitol) using the DynaMag-2 magnetic stand (Invitrogen). M^pro^-Avi (6 µg) was diluted to 1× selection buffer (50 mM Tris-HCl pH 7.5–150 mM sodium chloride–0.05% Tween-20–1 mM dithiothreitol–0.1 mg/mL sheared salmon sperm DNA), and an aliquot was collected as an input sample. The remaining Avi-tagged protein (5 µg) was immobilized to Dynabeads MyOne Streptavidin T1 paramagnetic beads at room temperature for 30 minutes. Flowthrough was collected, and beads were washed once with selection buffer. Beads were suspended to 1× selection buffer, a “beads” sample was collected, and the remaining bead suspension was heated at 95°C for 10 minutes. Beads were collected using a magnetic stand, and the solution was collected as a heated eluate sample. Beads were resuspended to selection buffer (heated beads). To visualize the relative amount of immobilized and eluted protein, the bead capture test samples were separated by SDS-PAGE. Samples were denatured and reduced using NuPAGE LDS Sample Buffer (4×) (Invitrogen) and NuPAGE Sample Reducing Agent (10×) (Invitrogen) by heating at 95°C for 5 minutes. Samples were separated on NuPAGE 4%–12% Bis-Tris Mini Protein Gels (Invitrogen) using a matched electrophoresis run chamber at 200 V for 35 minutes. Proteins on the gels were visualized using InstantBlue Coomassie Protein Stain (Abcam), destaining with distilled water.

### DECL affinity selection

Three rounds of affinity selection were performed using the third-generation DELopen DECL (WuXi AppTec; hits.wuxiapptec.com/delopen) according to the provider’s instructions. Before each round, M^pro^-Avi (5 µg) was immobilized to Dynabeads MyOne Streptavidin T1 paramagnetic beads at room temperature for 30 minutes in 1× selection buffer. Reversible small-molecule inhibitors were added at 20-µM concentration for the last 10 minutes of immobilization and included in DEL incubation mixtures at 20-µM final concentration. Covalent inhibitors at 5 µM were preincubated with an immobilized target for 10 minutes at room temperature but not added to the DEL incubation. The bead-immobilized target at a final concentration of 1.7 µM was incubated with the DELopen compound library in 1× selection buffer (50 mM Tris-HCl pH 7.5–150 mM sodium chloride–0.05% Tween-20–1 mM dithiothreitol–0.1 mg/mL sheared salmon sperm DNA) for 1 hour at room temperature with gentle rotation. Three wash cycles were performed with 1× selection buffer (50 mM Tris-HCl pH 7.5–150 mM sodium chloride–0.05% Tween-20–1 mM dithiothreitol) after each round. The compound release method was heating for 10 minutes at 98°C after each round. Samples collected after the third selection round were stored at −80°C before transfer to Wuxi AppTec for post-selection quality control and deep sequencing.

### Protease expression and purification

SARS-CoV-2 3CL protease (M^pro^) used for enzymatic assays and co-crystallization experiments was produced as previously described (25). The used construct contained nucleotide sequences corresponding to SARS-CoV-2 M^pro^ residues S1-Q306 (Chinese isolate, NCBI accession number YP_009725301). A detailed protocol is provided in the supporting information.

Avi-tagged SARS-CoV-2 M^pro^ (batch nr. MPRO_p009 GP-AVI) protease used for affinity selection of the DECL was kindly provided by Martin Walsh, Diamond Light Source, UK. A detailed protocol for the expression and purification of the enzyme is provided in the supporting information.

SARS-CoV-2 M^pro^ Washington strain (WA1, accession number MT246667), WT (M^pro^-WT), or carrying the E166V mutation (M^pro^-E166V) was expressed and purified as described in (26).

### Protein-ligand co-crystallization and x-ray data collection

Compounds SLL11, SLL12, and MP9 were added at 27-fold, 34-fold, and 13-fold excess, respectively, to a 5.2 mg/mL SARS-CoV-2 protease solution in 20 mM HEPES (pH 7.5) and 50 mM NaCl. Before co-crystallization, the non-dissolved ligand was spun down by centrifugation at 13,000 rpm for 30 seconds in a Hettich 200 R microcentrifuge.

For ligand SLL11, sitting-drop co-crystallization in 96-well Corning 3550 plates was performed by mixing equal amounts (150 + 150 nL) of ligand incubated protein with a solution consisting of 18% wt/vol PEG3350, 0.2 M potassium-thiocyanate, and 0.1 M bis-tris propane pH 8.5 using a mosquito robot. The first crystals of the SLL11 complex appeared after 53 days in the well solution and were harvested on day 98. For ligand SLL12, we performed hanging-drop co-crystallization in 24-well VDXm plates by manual mixing equal amounts (1 + 1 µL) of ligand incubated protein with a solution consisting of 20% wt/vol PEG3350, 0.2 M potassium-thiocyanate, and 0.1 M bis-tris propane pH 8.5. The first crystals of the M^pro^-SLL12 complex appeared after 35 days and were harvested at day 98. For ligand MP9, we performed sitting-drop co-crystallization in 96-well MRC plates by mixing equal volumes 150 + 150 nL of ligand incubated protein with a solution consisting of 18% wt/vol PEG3350, 0.2 M potassium-thiocyanate, and 0.1 M bis-tris propane pH 8.7 using a mosquito robot. The first crystals of the MP9 complex appeared after 5 days and were harvested on day 9. All crystals were cryo-protected by adding a cryo solution consisting of a solution supplemented with 15%–30% glycerol and 50 mM NaCl to the crystal droplet right before the crystals were picked up using Dual-Thickness MicroLoop and flash-frozen in liquid nitrogen.

X-ray diffraction data sets were collected at cryogenic 100 K temperature, using wavelength 0.9763 Å, at the MAX IV BioMAX beamline (27) in Lund Sweden (compounds: SLL11, PDB ID 9EO6, and SLL12, PDB ID 9EOR), and using wavelength 0.9795 Å, at Diamond Light Source (28), i04 beamline in Oxfordshire, UK (compound MP9, PDB ID 9EOX). All complex data sets crystallize in space group P1 with six SARS-CoV2 M^pro^ molecules in the asymmetric unit, and we collected 360 degrees of data using a single crystal for each complex and processed our data sets using the XDS (29) part of XDSAPP (30). The crystals diffracted better in some directions, and we, therefore, applied elliptical data truncation using the staraniso webserver (staraniso.globalphasing.org) leading to the capture of the best diffraction data at the expense of completeness in the highest resolution shells.

The structures were solved by molecular replacement using our in-house determined apo structure (7PFL) as a search model. For data scaling, molecular replacement, and refinement, software from the CCP4 suite (31) was used such as aimless (32), phaser (33), refmac5 (34), and coot (35). Refinement dictionaries for the ligands were generated from a ligand SMILES string using the grade web server (grade.globalphasing.org), Acedrg (36), and other recently developed ligand tools (37) in CCP4 to model the ligand–protein covalent bond. We used non-crystallographic symmetry restraints throughout refinement that ended when R/Rfree values did not improve further. The structures of compounds SLL11, SLL12, and MP9 have 97.0%/96.2%/96.5% residues favored, 2.7%/3.3%/2.7% residues allowed, and 0.3%/0.5%/0.8% residues in outlier regions of the Ramachandran plot. The SLL11, SLL12, and MP9 structures have been deposited in the protein data bank with accession 9EO6, 9EOR, and 9E0X, respectively, with data collection and refinement statistics presented in Table S1.

### *In vitro* enzymatic assay

All experiments were carried out as previously reported (25) in black flat-bottomed 96-well plates (Nunc, Thermo Fisher Scientific) in a final volume of 100 µL. Different concentrations of the compounds were incubated with recombinant SARS-CoV-2 protease (final concentration 100 nM) in assay buffer (20 mM HEPES pH 7.5, 0.01% Triton X-100) for 10 minutes at room temperature. The enzymatic reaction was started by the addition of the substrate Dabcyl-Lys-Thr-Ser-Ala-Val-Leu-Gln-Ser-Gly-Phe-Arg-Lys-Met-Glu-EDANS at a final concentration of 20 µM. The fluorescent emission was monitored every 60 seconds for 40 minutes using a Tecan Infinite M200 PRO plate reader (Tecan Trading AG, Switzerland) with the excitation wavelength set to 355 nm and the emission wavelength set to 538 nm.

For the initial screening, compound stocks (10 mM in 100% dimethyl sulfoxide [DMSO]) were diluted to 10 µM in assay buffer and then further diluted 10 times to the final concentration of 1 µM by transferring 10 µL in the assay wells (final volume of 100 µL). The final concentration of DMSO was kept at 0.01% (vol/vol) in all wells comprising the control wells. All compounds and controls were tested with triplicates.

For the SAR study, the compound’s IC_50_ were determined with a 12-point concentration series composed of one series of six 1:5 dilutions ranging from 4 to 0.00128 µM (final concentration in the well) and a second series of six 1:5 dilutions ranging from 2 to 0.00064 µM (final concentration in the well). The average IC_50_ and standard error of the means were calculated from two independent experiments with each compound concentration tested in triplicates. DMSO concentrations were always kept ≤0.04% (vol/vol) in all wells.

For resistance testing, MP6, MP9, and nirmatrelvir were tested at final concentrations ranging from 4 to 0.00005 µM (eight 1:5 serial dilutions). The recombinant SARS-CoV-2 M^pro^ (WA1, Washington strain) carrying the E166V mutation (M^pro^-E166V) was used at a final concentration of 500 nM. The same compounds were also tested at a concentration ranging from 100 to 0.78 µM (eight 1:2 serial dilutions) against the M^pro^-WT (final concentration of 100 nM) also from the WA1 strain for comparison. The average IC_50_ and standard error of the means were calculated from two independent experiments with each compound concentration tested in triplicates. DMSO concentrations were always kept to ≤0.04% (vol/vol) in all wells when testing the compounds against M^pro^-E166V and 1% (vol/vol) when testing the compounds against M^pro^-WT. The same concentration of DMSO was also present in control wells.

The relative fluorescence units (RFU) per second were plotted, and the initial velocities were calculated, normalized to the controls (untreated protease controls wells = 0% inhibition, control wells with no substrate = 100% inhibition), and expressed as % of enzyme activity inhibition. The IC_50_ was calculated by non-linear regression fitting of the normalized 12-point dose–response curve to the model “log(inhibitor) vs normalized response – variable slope” with equation *Y* = 100/1 + 10^(logIC^_^50^_^−^ *^X^*^)*HillSlope^.

The data analysis was conducted in GraphPad Prism (v.9.5., GraphPad Software, La Jolla, CA, USA).

### CPE-based antiviral assay

Calu-3 cells were grown in Dulbecco's modified Eagle medium (DMEM, Gibco, 41966029) supplemented with 10% fetal bovine serum (FBS, Gibco, 10500064) and 1× penicillin–streptomycin (Sigma-Aldrich, PA333) and incubated at 37°C, 5% CO_2_ atmosphere. The compound MP1 was tested at concentrations ranging from 20 to 0.156 µM (eight 1:2 serial dilutions). MP1 was tested in two independent experiments with each concentration tested in triplicates.

One day prior to the assay, Calu-3 cells were seeded at a density of 20,000 cells/well in a 96-well plate in a final volume of 100 µL of cell media (DMEM supplemented with 2% FBS and 1× penicillin−streptomycin, from now on referred to as DMEM-2). After overnight incubation (37°C, 5% CO_2_ atmosphere), cells were pretreated for 2 hours with 100 µL of DMEM-2 with CP-100356 (MedChemExpress, HY-108347) added at a final concentration of 4 µM. After 2 hours, the cell media containing CP-100356 was discarded; cells were washed with 100 µL of phosphate buffered saline (PBS) and infected with 200 plaque-forming units of SARS-CoV-2 (isolate from Sweden (38)) corresponding to a multiplicity of infection (MOI) ~0.01. After 1 hour, the cell media with the virus was discarded; cells were washed with 100 µL of PBS and treated by adding 100 µL of DMEM-2 containing different concentrations of MP1 and CP-100356 at a final concentration of 4 µM. After 48 hours, the cell media supplemented with MP1 and CP-100356 was substituted with 100 µL of fresh DMEM-2 to which 10 µL of a 5 mg/mL MTT [3-(4,5-dimethyl-2-thiazolyl)-2,5-diphenyl-2H-tetrazolium bromide, Sigma-Aldrich, M2128] solution in PBS was added. Following 4 hours of incubation, formazan crystals were solubilized overnight by adding 100 µL of a 10% SDS and 0.01 M HCl solution. Optical density (OD) at 570 and 690 nm was read using a Tecan Infinite M200 PRO plate reader (Tecan Trading AG, Switzerland). Throughout the assay, cell controls (not infected cells treated or not treated with MP1 at different concentrations) and infection controls (wells not treated with MP1 at different concentrations) were also treated with CP-100356 (4 µM), and DMSO concentration was kept constant to 0.25% (vol/vol) in all wells. OD readings at different wavelengths were subtracted, the resulting values were normalized to the controls, and EC_50_ were determined by non-linear regression analysis using GraphPad Prism (vr.9.5, GraphPad Software, La Jolla, CA, USA).

### Virus yield reduction assay

Caco-2 cells were grown in DMEM (Gibco, 41966029) supplemented with 10% FBS (Gibco, 10500064) and 1× penicillin–streptomycin (Sigma-Aldrich, PA333) and incubated at 37°C, 5% CO_2_ atmosphere. The compound MP1 was tested at 5, 0.5, and 0.05 µM (eight 1:2 serial dilutions). MP1 was tested in two independent experiments with each concentration tested in triplicates.

One day prior to the assay, Caco-2 cells were seeded at a density of 20,000 cells/well in a 96-well plate in a final volume of 100 µL of cell media (DMEM supplemented with 2% FBS, 1× penicillin−streptomycin). After overnight incubation (37°C, 5% CO_2_ atmosphere), cells were infected with 200 plaque-forming units of SARS-CoV-2 [isolate from Sweden (38)] corresponding to a MOI ~0.01. After 1 hour, the cell media with the virus was discarded; cells were washed with 100 µL of PBS and treated by adding 100 µL of cell media (DMEM supplemented with 2% FBS, 1× penicillin−streptomycin) containing MP1 at different concentrations. After 48 hours, supernatants were collected (100 µL) and mixed with 300 µL of TRIzol LS (Invitrogen, Thermo Fisher Scientific, Waltham, MA). The viral RNA was extracted using the Direct-zol-96 RNA Kit (Zymo Research, Irvine, CA) according to the manufacturer’s protocol. The extracted viral RNA was quantified by RT-qPCR using primers (Thermo Fisher Scientific, Waltham, MA) previously described (39) and the SuperScript III OneStep RT-PCR System with Platinum Taq DNA Polymerase kit (Invitrogen, Thermo Fisher Scientific, Waltham, MA). Target E: forward primer 5′-ACAGGTACGTTAATAGTTAATAGCGT-3′; reverse primer 5′ GTGTGCGTACTGCTGCAATAT-3′; and the probe 5′-FAM-CACTAGCCATCCTTACTGCGCTTCG-TAMRA-3′. The reaction mixture contained 12.5 µL of reaction buffer (a buffer containing 0.4 mM of each dNTP, 3.2 mM Mg_2_SO_4_), 0.5 µL of SuperScript III RT/Platinum Taq Mix, 0.5 µL of each primer (10 µM stock concentrations), 0.25 µL probe (10 µM stock concentration), 2.4 µL of 25 mM magnesium sulfate, 3.35 µL of nuclease-free water, and 5 µL of RNA template. The RT-qPCR assay was performed on a CFX96 Touch Real-Time PCR Detection System (Bio-Rad Laboratories, Hercules, CA) under the following conditions: reverse transcription at 55°C for 30 minutes and 95°C for 3 minutes, followed by 45 cycles of denaturation at 95°C for 15 seconds, extension at 57°C for 30 seconds, and collection of the fluorescence signal at 68°C for 30 seconds.

All samples were run in triplicate. The corresponding number of copies for each Ct was calculated from a standard curve prepared with synthetic DNA gene fragments (gBLOCKs; IDT, San Jose, CA) with a 5-base-pair deletion in the amplified regions of the viral genome diluted in deionized, nuclease-free water to concentrations of 103–105 copies/µL. The 5 base pairs were deleted to be able to distinguish between viral RNA and gBLOCKs during sequencing. The limit of detection for both genes was 101 copies/µL. The RFU data were obtained from the CFX Maestro Software (Bio-Rad CFX Maestro for Mac 1.1 Version 4.1.2434.0214, Bio-Rad Laboratories, Hercules, CA). Quantified viral RNA from infected wells treated with different concentrations of the compounds were normalized to the controls using GraphPad Prism (vr.9.5, GraphPad Software, La Jolla, CA).

### Compound synthesis

All compounds were synthesized by solid-phase peptide synthesis on 2-chlorotrityl chloride resin (2CTC) using different Fmoc-protected natural and unnatural amino acids. All compounds prepared were either dipeptides or tripeptides terminated by a carboxylic acid as a capping agent at the N-terminus. Below is a general synthetic procedure describing the preparation of a carboxylic acid-capped tripeptide. The syntheses of the corresponding capped dipeptides were carried out in an analogous fashion using less amino acid coupling.

The 2CTC resin (63 mg, 0.1 mmol, 1 equiv.) was added to a 3-mL syringe with a frit, after which a solution of Fmoc-protected amino acid #1 (0.2 mmol, 2 equiv.) and *N*,*N*-diisopropylethylamine (DIPEA, 0.05 mL, 0.30 mmol, 3 equiv.) in dichloromethane (DCM, 1 mL) was prepared and subsequently aspirated into the syringe. The resulting mixture was agitated at room temperature for 2 hours to allow for the coupling of amino acid #1 to the resin, after which the reaction solution was ejected and the 2CTC resin was washed with dimethyl formamide (DMF, 2 × 1 mL) under agitation for 30 seconds. To deactivate the remaining 2CTC functionalities on the resin, a solution of DCM/methanol/DIPEA (ratio 85:15:5, 1.05 mL) was aspirated into the syringe followed by agitation for an additional 15 minutes. The 2CTC resin was then washed with DMF (2 × 1 mL) and DCM (3 × 1 mL), before being subjected to subsequent Fmoc deprotection. Removal of the Fmoc group was done by treating the 2CTC resin with piperidine in methanol (80%, 1 mL) for 20 minutes under agitation at room temperature. The reaction solution was then ejected from the syringe, and the 2CTC resin was carefully washed using DMF (3 × 1 mL), methanol (2 × 1 mL), DCM (2 × 1 mL), and DMF (2 × 1 mL) before coupling of the next amino acid. The following method was used for the coupling of amino acid #2 (0.15 mmol) and was subsequently repeated for the coupling of amino acid #3 (0.15 mmol) and the capping carboxylic acid (0.40 mmol with 3.8 equiv. HATU). The 2CTC resin was treated in a 3-mL syringe with a frit with a solution of amino acid #2 (0.15 mmol) and DIPEA (0.07 mL, 0.4 mmol, 4 equiv.) in DMF (0.6 mL). To this mixture was then aspirated a solution of HATU (72 mg, 0.19 mmol, 1.9 equiv.) in DMF (0.4 mL), and the corresponding solution was agitated at room temperature for 45 minutes, after which it was washed carefully with DMF (3 × 1 mL), methanol (2 × 1 mL), DCM (2 × 1 mL), and DMF (2 × 1 mL). Following the coupling of amino acids #2 and #3, removal of the Fmoc group was done by treating the 2CTC resin with piperidine in methanol (80%, 1 mL) for 20 minutes under agitation at room temperature. The reaction solution was then ejected from the syringe, and the 2CTC resin was carefully washed using DMF (3 × 1 mL), methanol (2 × 1 mL), DCM (2 × 1 mL), and DMF (2 × 1 mL) before coupling of the next amino acid (or carboxylic acid in the last reaction). After completing synthesis, the target peptides were cleaved from the 2CTC resin using a mixture of hexafluoroisopropanol/DCM (700 µL/300 µL), followed by agitation for 10 minutes. The final product (purity >95%) was isolated after preparative high-performance liquid chromatography (HPLC) purification using the mobile phase: 20%–60% MeCN in H_2_O with 0.05% formic acid over 8 CV with a flow of 30 mL/minute.

### Synthesis of the Dabcyl-KTSAVLQSGFRKME-EDANS substrate

EDANS NovaTag resin (200 mg, 0.6 mmol/g) was preswelled in DMF. Fmoc-Glu-OH (3 eq.), HATU (3 eq.), and DIPEA (6 eq.) in DMF were thereafter added to the resin. The reaction mixture was gently agitated in an overhead shaker for 60 minutes. The resin was washed several times with DMF whereafter the Fmoc protection was removed with 4% DBU (1,8-diazabicyclo[5.4.0]undec-7-ene) in DMF followed by washings with DMF, isopropanol, methanol, H_2_O, and DCM. The coupling procedure was repeated for the next coming Fmoc-amino acids in the following order: Fmoc-Met, Fmoc-Lys, Fmoc-Phe, Fmoc-Gly, Fmoc-Ser, Fmoc-Asn, Fmoc-Leu, Fmoc-Val, Fmoc-Ala, Fmoc-Ser, Foc-Thr, and Fmoc Lys. In the final step, Dabcyl was introduced by adding Dabcyl acid (3 eq.), HATU (3 eq.), and DIPEA (6 eq.) in DMF followed by agitation for 60 minutes. Final washing was done using DMF, iPrOH, MeOH, H_2_O, and DCM. The final labeled peptide was cleaved from the resin using TFA:TES 95/5. The filtrate was collected, and the crude product was isolated by evaporation. The final product (purity 99%) was isolated after preparative HPLC purification using the mobile phase: 0.1% TFA acetonitrile and 0.1% TFA in water (15–45%).

## RESULTS

### DNA-encoded chemical library screening

**Fig 1:**
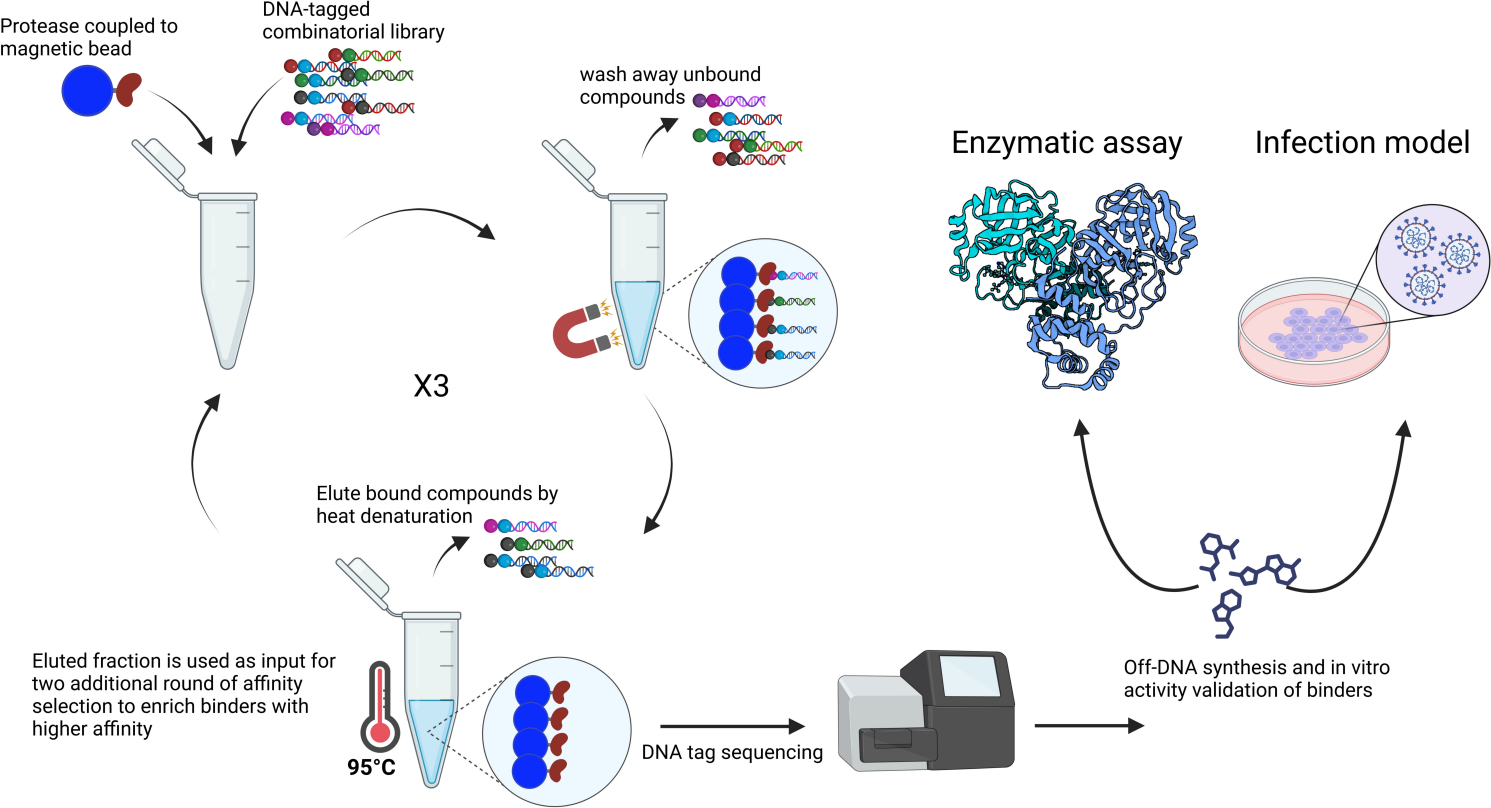
Workflow of hit identification and validation using DNA-encoded chemical libraries. Novel inhibitors of SARS-CoV-2 M^pro^ were identified from the DELopen library containing 4.2 billion DNA-tagged compounds. After three rounds of affinity selection, the unique DNA-tag of binders was sequenced allowing the identification of highly enriched compounds with their numeric building block codes. The molecular structures of the most promising compounds were subsequentially disclosed by WuXi AppTec; four compounds were synthetized off-DNA and tested to confirm their *in vitro* inhibitory activity against M^pro^ and antiviral activity against SARS-CoV-2 in cell-based assays.

**Fig 2:**
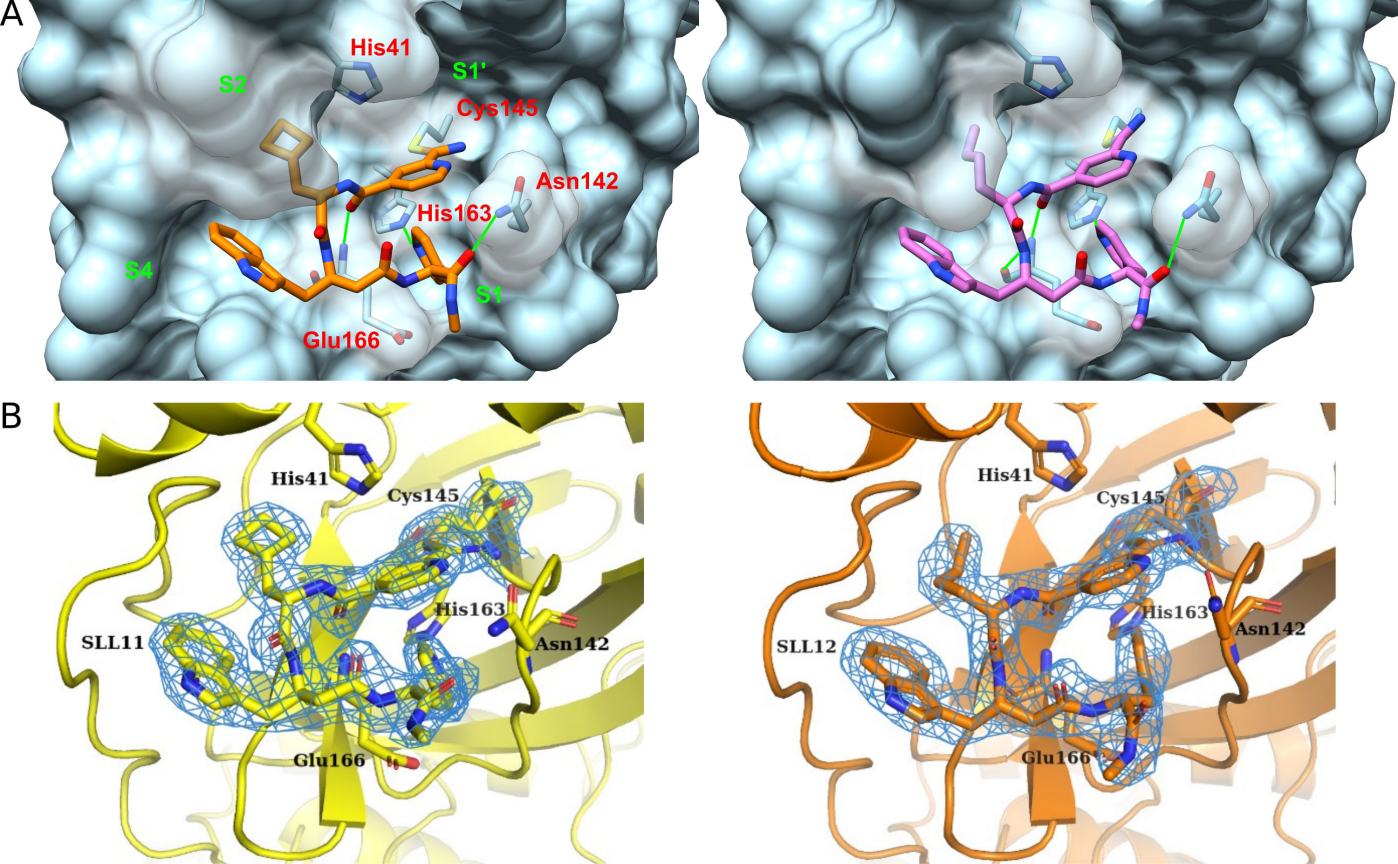
Compound’s general structure and interactions made with SARS-CoV-2 M^pro^. (**A**) Crystal structures of compounds SLL11 (PDB ID 9EO6), shown in orange, and SLL12 (PDB ID 9EOR), shown in purple, bound to SARS-CoV-2 M^pro^ active site. The two residues forming the enzyme’s catalytic dyad (His41 and Cys145) and the residues found interacting with SLL11 and SLL12 are shown in red. The subsites of M^pro^ active site and predicted hydrogen bonds are labeled and drawn in green, respectively. (**B**) Electron density maps (2fo-fc) covering the ligands SLL11 (yellow) and SLL12 (orange) contoured at 1.2 sigma level.

The *in vitro* affinity selection of binders from DELopen library (WuXi AppTec) was performed against recombinant avi-tagged SARS-CoV-2 M^pro^ coupled to paramagnetic beads coated with streptavidin (Fig. 1). The efficiency of M^pro^ coupling to the paramagnetic beads and the activity of M^pro^ bound to the magnetics beads were tested prior to the selection experiments, ensuring that the protease was stably bound to the beads while retaining its active conformation (Fig. S1 and S2). The selection of binders was performed with or without the addition of SARS-CoV-2 M^pro^ inhibitors X77 (20 µM) or GC376 (5 µM), which are known to bind and block the active site of M^pro^. Therefore, compounds selected in the presence of X77 or GC376 were regarded as possibly binding to an allosteric pocket. The selection was also performed against empty beads to filter out promiscuous binders of no interest. After three rounds of selection, four highly enriched binders of which one (SLL11) was a possible allosteric inhibitor were identified by next-generation sequencing (NGS) and selected for off-DNA synthesis (NGS and synthesis of compounds were provided by WuXi AppTec). The inhibitory activity of the synthesized compounds against recombinant SARS-CoV-2 M^pro^ (Table 1) was confirmed using a FRET-based enzymatic assay. Three out of the four synthetized compounds were active with submicromolar IC_50_ ranging from 30 to 140 nM. The two most enriched binders against M^pro^, SLL11 and SLL12, were also the most potent (SLL11 IC_50_ = 30 nM, SLL12 IC_50_ = 53 nM) while the least enriched binder (SLL08) was not active at the tested concentrations (IC_50_ >50 µM). The active compounds displayed high selectivity for SARS-CoV-2 M^pro^ and had no off-target activity against human cathepsin S when tested up to a concentration of 50 µM (Fig. S3). The two most potent compounds were synthesized and co-crystallized with M^pro^ to determine their binding pose and binding site. SLL11 and SLL12 are peptide-like compounds (Fig. 2) composed of three non-natural amino acids of which P1′ present an electrophilic group. Both compounds were found to bind to the M^pro^ active site with similar binding poses proving that SLL11 was not an allosteric inhibitor despite being selected and enriched also in the presence of the M^pro^ inhibitors X77 and GC376. This was not surprising as SLL11 and SLL12 shared three out of four groups.

**TABLE 1:** List of compounds selected from DECLs and relative *in vitro* activity

Compound	Enrichment score^*a*^				IC_50_ (µM)	
	M^pro^	M^pro^ +X77	M^pro^ +GC376	Beads only	M^pro^	Cathepsin S
SLL07	135,654	0	0	0	0.14	>50
SLL08	11,444	0	676	0	>50	>50
SLL11	515,485	20,997	206,982	0	0.03	>50
SLL12	488,354	0	0	0	0.053	>50
___________________________________________________________________________________ 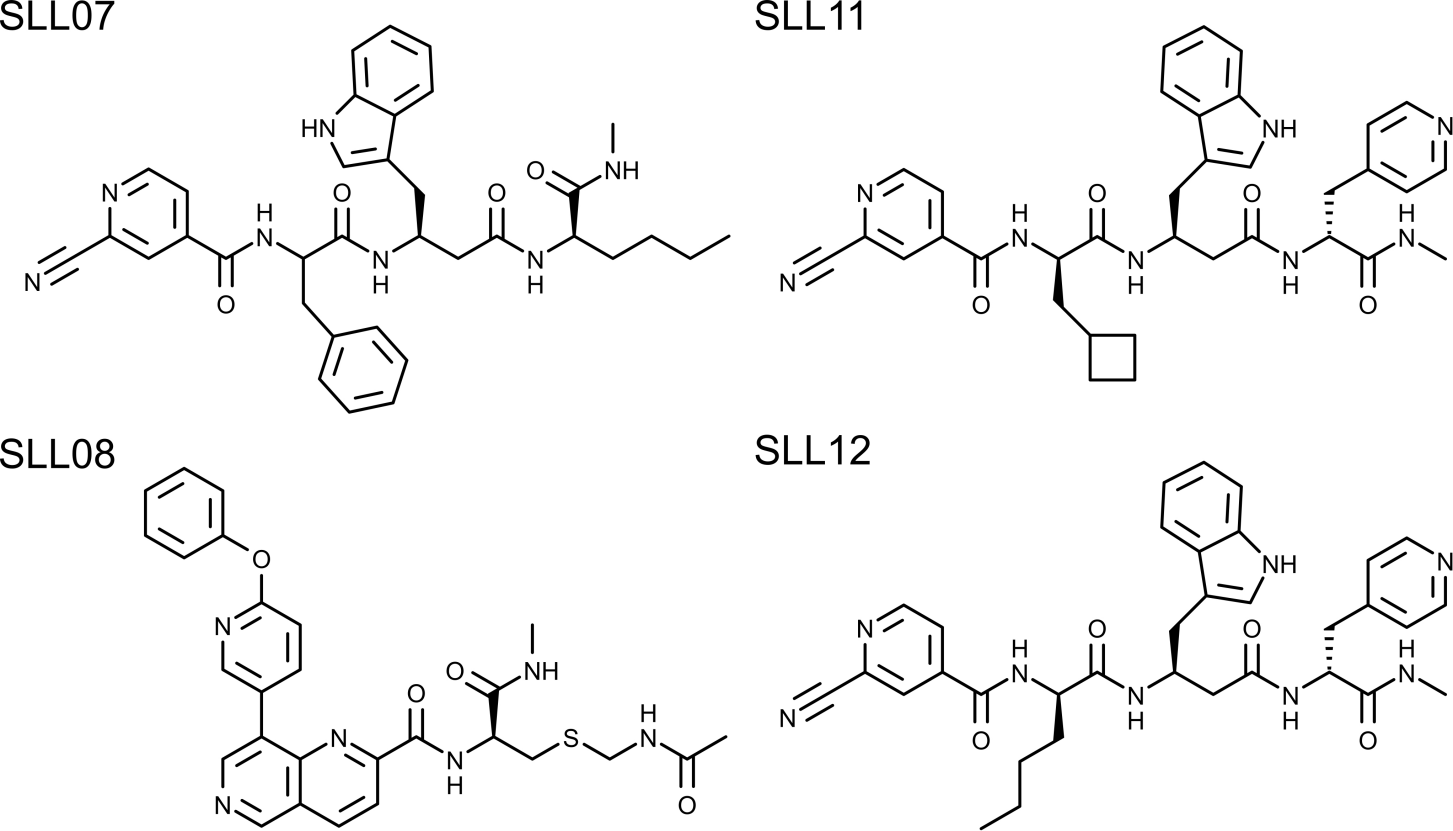						

^*a*^ The enrichment score quantifies how abundant a compound was after selection, e.g., a compound with enrichment score = 100 was 100 times more abundant than average.

The compounds interacted with M^pro^ mainly via a covalent bond formed between the electrophilic P1′ group and the catalytic Cys145 close to the S1′ subsite, and a hydrogen bond formed between the P3 group and His163 in the S1 subsite, while P2 and P3 hydrophobic residues occupied the S2 and S4 subsites. Hydrogen bonds were also formed between the backbone of the compounds and Glu166 and between the C-terminal amine of SLL11 and M^pro^ Asn142 side chain.

### Structure activity relationship study

Compound MP1 was obtained substituting the C-terminus of compound SLL11 (IC_50_ = 30 nM) with a carboxylic acid group to increase the compound solubility (Table 2). This structural difference caused no loss of activity and most likely did not affect its binding mode (Fig. S4); thus, compound MP1 (IC_50_ = 25 nM) was used as a starting point and reference for the design of analogs and comparison of inhibitory activity (Table 2). The inhibitory activity of analogs against recombinant SARS-CoV-2 M^pro^ was first screened at a concentration of 1 µM, and analogs inhibiting M^pro^ activity by 80% or more were further tested to determine their IC_50_ (dose–response curves are shown in Fig. S5).

**TABLE 2:** List of MP1 analogs and relative inhibitory activity against SARS-CoV-2 M^pro^

Compound	M^pro^ inhibition %^*a*^ (comp. conc. 1 µM)	IC_50_ ± SEM (µM)^*b*^	Compound	M^pro^ inhibition %^*a*^ (comp. conc. 1 µM)	IC_50_ ± SEM (µM)^*b*^
MP1	97% ± 4.9 SD	0.025 ± 0.001	MP9	86.6% ± 1.1 SD	0.139 ± 0.049
MP2	2.2%	n.d.	MP10	95.7% ± 1.3 SD	0.071 ± 0.007
MP3	30.4%	n.d.	MP11	90.7% ± 1.6 SD	0.069 ± 0.004
MP4	38% ± 16.2 SD	n.d.	MP12	96.5% ± 4.5 SD	0.106 ± 7^*c*^
MP5	8.1%	n.d.	MP13	94.2% ± 5.7 SD	0.047 ± 0.006
MP6	17.8%	n.d.	MP14	98.2% ± 2.6 SD	0.045 ± 0.009
MP7	100%	0.024 ± 0.0004	MP15	66.8%	n.d.
MP8	18.2%	n.d.	MP16	94.7% ± 3.4 SD	0.033 ± 0.003
_____________________________________________________________________________________________________________________________ 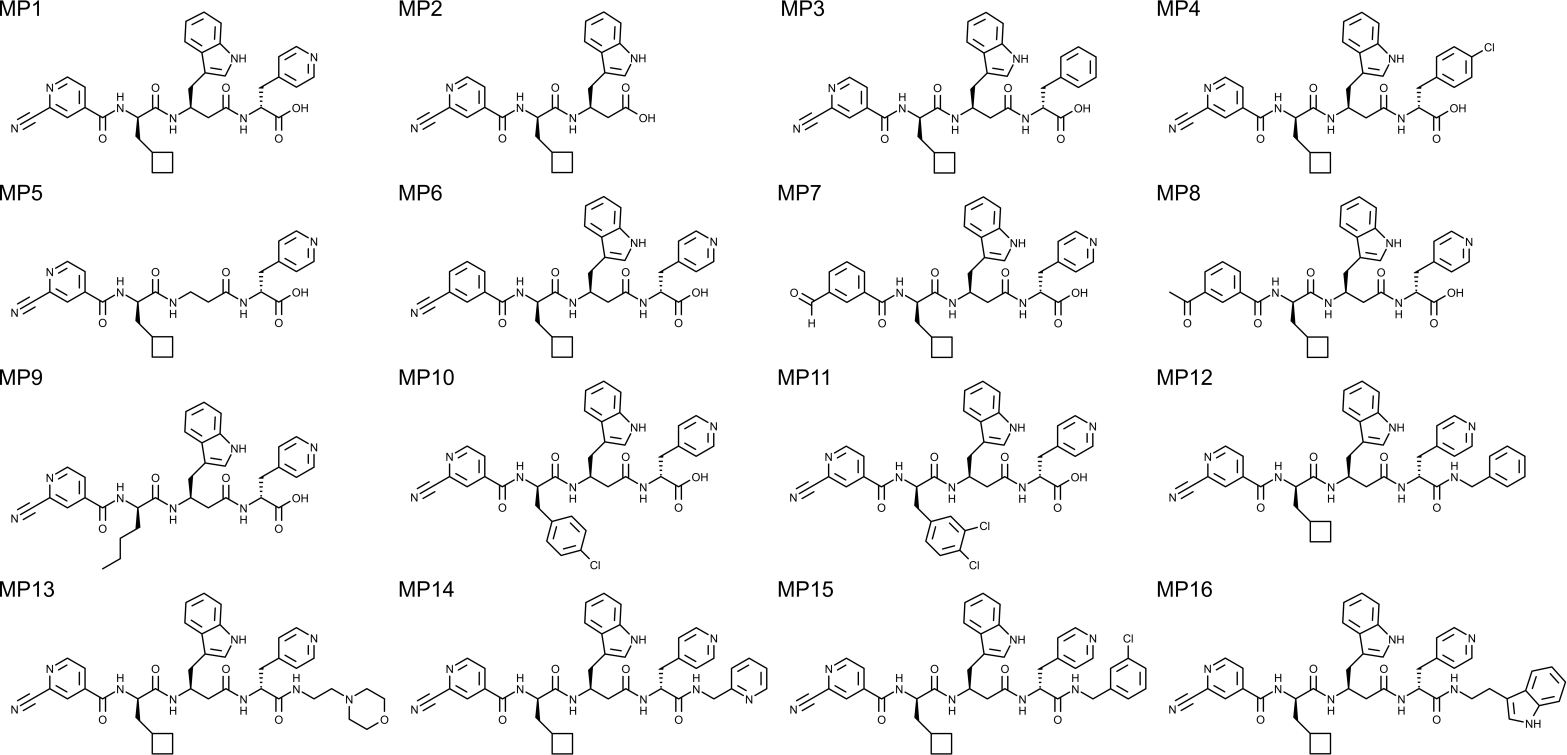					

^*a*^ Compounds were tested at a concentration of 1 µM with triplicates (*n* = 3 replicates). Average inhibition of M^pro^ ± standard deviation (SD) is shown only for compounds that were tested in two independent experiments.

^*b*^ Average IC_50_ and standard error of the mean (SEM) were calculated from two independent experiments where each compound’s concentration was tested in triplicates (*n* = 3 replicates).

^*c*^ The IC_50_ of compound MP12 was determined from a single experiment where each compound’s concentration was tested in triplicates (*n* = 3 replicates); SD is reported instead of SEM.

We assumed that the covalent bond formed between the N-terminal electrophilic group and Cys145 was essential for MP1 activity and started designing analogs with modification at the C-terminus of the molecule. First, analogs presenting variations of the P3 and P2 residues were designed to assess if the size of MP1 could be reduced while retaining activity. As observed from the crystallographic binding poses, the P3 residue occupies the S1 subsite of M^pro^ and forms a hydrogen bond with His163. Therefore, removing the P3 residues (compound MP2) or substituting P3 with groups lacking a hydrogen bond acceptor (compounds MP3 and MP4) were poorly tolerated, reducing the compound inhibitory activity to ~2% and ~30%, respectively. Removing the side group of the P2 residue while maintaining the portion of the backbone connecting P3 to the P1 residue (compound MP5) also sharply reduced the inhibitory effect to ~8%. Since both P3 and P2 groups were essential for binding and attempts to shorten MP1 were unsuccessful, we next assessed the contribution of the P1′ electrophilic group to the binding of MP1 to the active site of SARS-CoV-2 M^pro^. As expected, substituting the P1′ residue with a less reactive analog (compound MP6) was detrimental (~18% residual inhibitory activity) while a highly reactive aldehyde (compound MP7, IC_50_ = 24 nM) was equipotent to the original electrophile group of MP1. A bulkier electrophile group at P1′ (compound MP8) also reduced the inhibitory activity against M^pro^ to ~18%. Lastly, the analogs MP9, MP10, and MP11 were designed to evaluate variants of the P1 residues. Exchanging the hydrophobic P1 group to other hydrophobic aliphatic (MP9 IC_50_ = 139 nM) or planar aromatic (MP10 IC_50_ = 71 nM and MP11 IC_50_ = 69 nM) groups reduced the IC_50_ of the analogs up to fourfold. However, all the analogs maintained a submicromolar activity with IC_50_ in the low nanomolar range.

Overall, all residues constituting MP1 seemed to be well optimized, so we next explored the post-modification of the C-terminus with various amide groups. Analogs with polar amide substituents (compounds MP13 IC_50_ = 47 nM, MP14 IC_50_ = 45 nM, and MP16 IC_50_ = 47 nM) were approximately twofold more potent than analogs with a non-polar amide group (MP12 IC_50_ = 106 nm), suggesting that this substituent might take part in hydrogen bonding with residues located near the S1 subsite of M^pro^.

### Antiviral activity of MP1 against SARS-CoV-2 in cell-based assays

**Fig 3:**
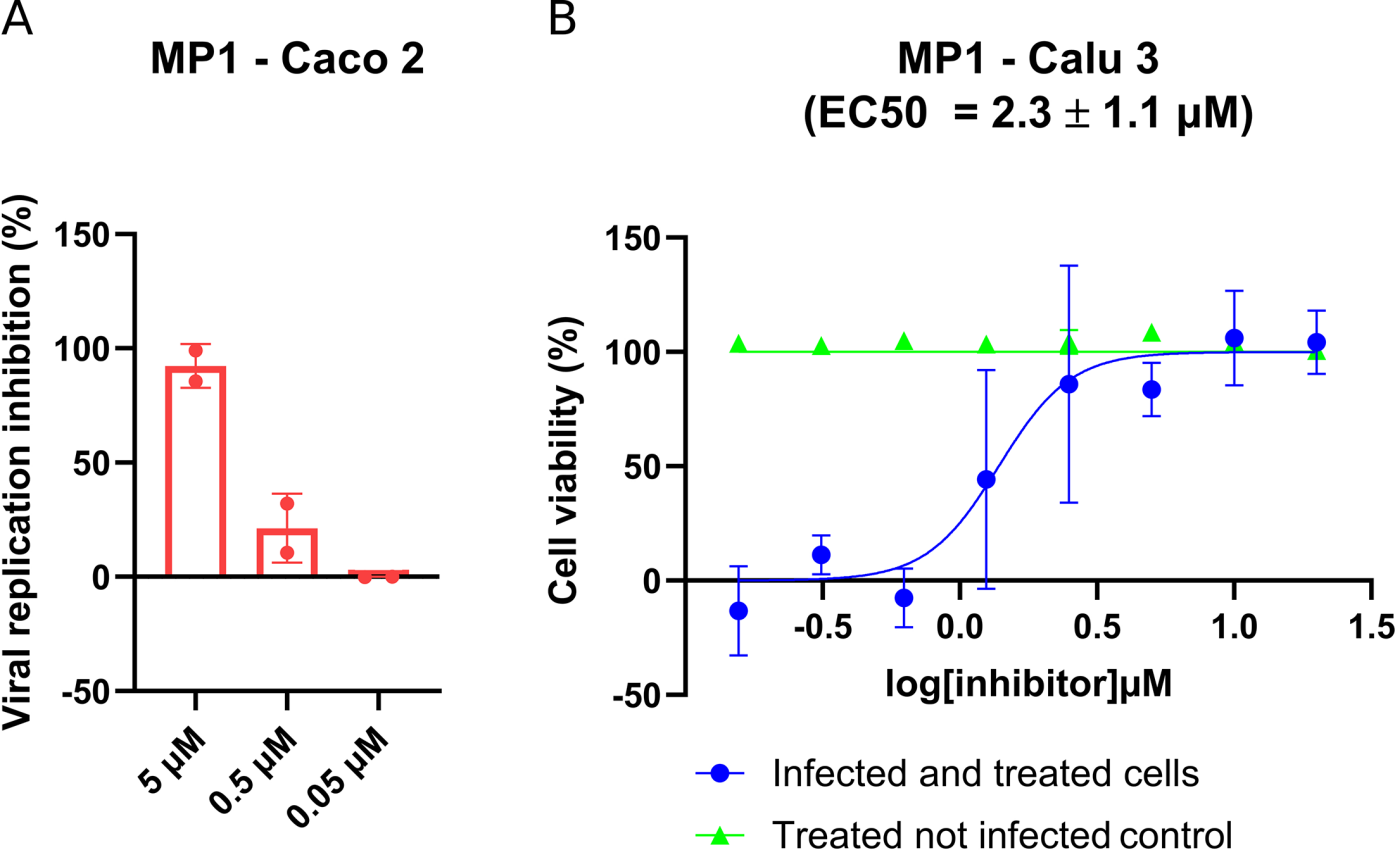
Antiviral effect of MP1 against SARS-CoV-2. (**A**) The inhibitory effect on viral replication was assessed using a yield reduction assay. Caco-2 cells were infected with SARS-CoV-2 (MOI 0.01) and treated with 5, 0.5, or 0.05 µM of MP1. Supernatants were collected 48 hours post-infection, and viral copy numbers per milliliter were quantified by RT-qPCR. The reduction in viral copy number per milliliter from two independent experiments is reported as inhibition of viral replication (%) ±SD. (**B**) Inhibitory effect on CPE development induced by SARS-CoV-2 infection. Calu-3 cells were infected (MOI 0.01) and treated with different concentrations of MP1; 48 hours post-infection cell viability was assessed by the MTT assay. The average EC_50_ from two independent experiments is reported ±the standard error of the mean.

MP1 was chosen to test the antiviral activity of the novel scaffold discovered in this study. The compound cell permeability first evaluated Caco-2 cells (human colon epithelial cells) that were cultured on a permeable filter. MP1 showed good apical to basolateral (A–B) permeability (*P*app A–B = 5.4 × 10^6^ ± 3.6 × 10^6^ cm/s) and lower basolateral to apical (B–A) permeability (*P*app B–A = 2.5 × 10^6^ ± 0.6 × 10^7^ cm/s) with no observable efflux of the compound (*P*app B–A/*P*app A–B = 0.5). Accordingly, MP1 was active in Caco-2 cells and showed dose–response inhibition of the viral replication measured by RT-qPCR (Fig. 3A). Since Caco-2 cells are highly permissive to SARS-CoV-2 infection but do not develop cytopathic effects (CPE), we tested the capacity of MP1 to inhibit CPE development and increase cell viability in infected Calu-3 cells (human lung epithelial cells). Unexpectedly, MP1 had no protective effect on infected Calu-3 at concentrations as high as 20 µM (data not shown). Based on previous studies reporting the P-glycoprotein (P-gp)-mediated efflux of M^pro^ peptide-like inhibitors (12, 40, 41), we assumed MP1 is a substrate of P-gp. When tested in combination with the P-gp inhibitor CP-100356 (4 µM), MP1 showed a dose-dependent inhibition of CPE development with EC_50_ = 2.3 ± 1.1 µM and an observable cytotoxic effect (Fig. 3B).

### Effect of the E166V M^pro^ variant on MP1 and MP7 inhibitory activity

**Fig 4:**
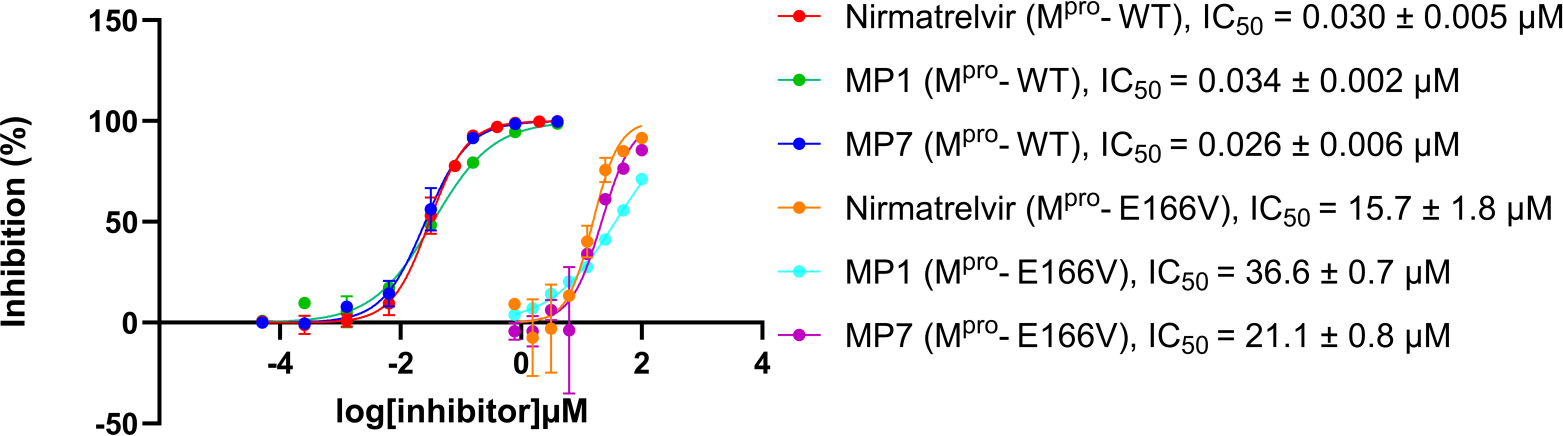
Effect of the E166V variant on the inhibitory activity of MP1 and MP7 compounds. Dose–response curves of MP1 and MP7 tested against recombinants wild-type (M^pro^-WT) and mutated M^pro^ carrying the E166V (M^pro^-E166V). IC_50_ values are reported as average ± SEM from two independent experiments performed with triplicates. Nirmatrelvir was also tested for comparison.

The inhibitory activity of compounds MP1 and MP7 was tested against recombinant M^pro^ carrying the E166V (M^pro^-E166V) variant, known to confer resistance against nirmatrelvir. Compounds MP1 and MP7 and nirmatrelvir, used as a reference, had comparable IC_50_ in the low nanomolar range against the recombinant wild-type M^pro^ (M^pro^-WT). The inhibitory activity of the three compounds was significantly decreased to 36.6 µM (MP1), 21.1 µM (MP7), and 15.7 µM (nirmatrelvir) and against M^pro^-E166V (Fig. 4).

## DISCUSSION

In this study, we report the identification of novel inhibitors of SARS-CoV-2 M^pro^ from the commercially available “DELopen” DECL. Three potent compounds (SLL07, SLL11, and SLL12) with IC_50_ in the low nanomolar range were directly identified through the cost-efficient affinity screening of 4.2 billion combinatorial molecules. The selected peptide-like compounds were formed by three unnatural amino acids capped at the N-terminus by a carboxylic acid derivative carrying a reactive electrophilic group. Of these three compounds, SLL11 (IC_50_ = 30 nM) was the most potent one followed by SLL12 (IC_50_ = 53 nM). SLL11 and SLL12 were structurally similar sharing two out of three unnatural amino acids, and both were found to bind to the M^pro^ active site. Crystallographic studies of SLL11 and SLL12 binding poses showed that the side groups of all three residues (P1, P2, and P3) and the N-terminal cap occupied one of the major subsites of the M^pro^ active site (S1′, S1, S2, and S4) with a novel arrangement of the peptide-like backbone to fit the binding site. The backbone of compounds SLL11 and SLL12 in the crystal structures assumed an unprecedented semi-cyclic conformation with the N-terminus and C-terminus stacked and possibly forming a T-shaped non-bonded π-π interaction (Fig. 2). In this conformation, the N-terminal electrophilic group binds Cys145 close to the S1′ pocket while the C-terminal pyridine group binds in the S1 pocket. This C-terminus to N-terminus orientation is markedly different from the orientation of the natural substrate of M^pro^ (42) and its substrate-derived inhibitors, which usually have the C-terminal amino acid positioned in the S1 or S1′ position while the remaining peptide chain and N-terminus extend toward the left side of the binding site. This unusual confirmation becomes more evident when the binding poses of SLL11 and nirmatrelvir bound to the M^pro^ of SARS-CoV-2 are compared as shown in Fig. S6 and is ascribed to the presence of unnatural D-amino acids and β-amino acids in the structure of SLL11 that enables it to adopt a U shape and be able to bind the S1 and S1′ subsites with its C- and N-terminus.

As stated above, SLL11 and SLL12 covalently bound to SARS-CoV-2 M^pro^; this means that the bond formed between the compounds’ nitrile group and the catalytic Cys145 was repeatedly reversed during the elution step (10-minute incubation step at 98°C) after each round of selection. Compounds containing aldehyde groups have also been eluted by applying heat (10-minute incubation at 80°C) as reported by Chamakuri et al. (20), confirming that the affinity screening of DECL can be used to identify reversible covalent inhibitors.

The affinity screening of the DECL was also performed with the addition of inhibitors known to bind and block the active site of M^pro^ to identify compounds that might bind to allosteric pockets. While both SLL11 and SLL12 were proven to bind to the active site of M^pro^, SLL11 could also get enriched during the three rounds of the selection process when the competitive inhibitor X77 was added or the reversible covalent inhibitor GC376 (43) was preincubated (10 minutes) with M^pro^. This proves that SLL11 was able to outcompete to some degree both X77 (IC_50_ = 2.8 µM) (11) and GC376 (IC_50_ = 0.15 µM) (44) used at final concentrations 14 times (20 µM) and 3.6 times (5 µM) higher than the concentration of the protease (~1.4 µM) respectively. Therefore, it is recommended to use the most potent available inhibitors, ideally with activity in the low nanomolar range, at high concentrations when it is important to differentiate between compounds binding or not to a specific site on the target of interest. Using irreversible covalent inhibitors instead of reversible covalent inhibitors like GC376 might also improve the discriminatory performance of the affinity selection process.

Attempts to further improve the compounds’ potency by systematically varying one of the building block residues resulted in the design of several analogs with IC_50_ lower than 100 nM. However, SLL11 (and its close analog MP1) remained the most potent inhibitor (IC_50_ = ≤30 nM) proving that the screening of large combinatorial libraries can identify well-optimized compounds without the need for several iterative cycles of medicinal chemistry.

Reducing the molecular weight, thus, improving the physiochemical properties of the compounds proved to be difficult. The inhibitory activity of analogs with a less reactive electrophile at P1′ was almost completely abrogated, suggesting that a covalent interaction with M^pro^ was essential. Co-crystallization of SLL11 and SLL12 with SARS-CoV-2 M^pro^ confirmed that the N-terminal nitrile electrophilic group formed a covalent bond with Cys145 of enzyme’s catalytic dyad as reported for several other peptide-like inhibitors of M^pro^ (12, 41, 44, 45). Likewise, removing the whole P3 residue or substituting the P2 β-tryptophan with a β-alanine lacking a side group was not possible without causing a drop in compound inhibitory activity. P3 formed an essential hydrogen bond with S1 His163 that mimics the interaction with the glutamine residue of its natural substrate (46). The side group of the P2 residue did not form any hydrogen bond with residues in the S4 pocket but was still essential for activity, suggesting that P2 contributes with more than just connecting P3 to the remaining part of the molecule.

When tested in cell-based assays, MP1 showed antiviral activity against SARS-CoV-2 in the low micromolar range in both Caco-2 and Calu-3 (EC_50_ = 2.3 ± 1.1 µM), two cell lines commonly used as infection models for SARS-CoV-2. However, while no efflux was observed in the Caco-2 permeability test, the addition of CP-100356 was necessary to prevent MP1 efflux mediated by P-glycoprotein (P-gp) in Calu-3 cells. This finding supports a previous report of Calu-3 cells expressing P-gp at a higher level than Caco-2 cells (47). The relatively low activity of MP1 in the cell-based assay could be explained by the compound’s sensitivity to proteolytic degradation as both SLL11 and SLL12 showed low metabolic stability in human and mouse microsomes (Table S2). Cyclization could improve the metabolic stability of the peptide-like compounds reported in this study and will be subject to further studies in our lab.

Finally, the *in vitro* inhibitory activity of MP1 and MP7 was evaluated against recombinant M^pro^ carrying the variant E166V that cause high fold resistance to nirmatrelvir (26, 48, 49). Nirmatrelvir had a 523-fold resistance when tested against the M^pro^-E166V in our FRET-based enzymatic assay. The observed increase in nirmatrelvir’s IC_50_ against SARS-CoV-2 M^pro^ was comparable to the 473-fold increase reported by Lan et al. in a similar enzymatic assay (26). The compounds MP1 and MP7 were also subject to decreased inhibitory activity and had 1,076- and 811-fold resistance, respectively, when tested against M^pro^-E166V. The dramatic effect of E166V mutation on nirmatrelvir activity was shown to be due to steric clashes in S1 preventing the formation of hydrogen bonds between nirmatrelvir’s γ-lactam ring, residues His163 and possibly also Asp166 itself, and Phe140. As a result of the disrupted key interaction in S1, the nitrile warhead is moved out of position making it more difficult to interact with Cys145 (26, 49).

MP1 and MP7 also interact with residue His163 through the hydrogen bonding and Cys145 with their electrophilic warheads at P1′. It is, therefore, plausible that similar dynamics are the cause of the reduced activity observed *in vitro* for compounds MP1 and MP7.

In conclusion, the affinity-based selection of an ultralarge DECL resulted in the direct identification of novel peptide-like inhibitors of SARS-CoV-2 M^pro^ with *in vitro* inhibitory activity in the low nanomolar range against recombinant M^pro^ and inhibitory activity in the low micromolar range in cell-based assays. More importantly, these compounds assume a conformation never reported before when bound to the active site of M^pro^, providing new venues for the development of coronaviruses’ M^pro^ inhibitors. Therefore, as demonstrated herein, the affinity screening of DECL and the intrinsic combinatorial nature of DECL allow the rapid exploration of large chemical spaces and could greatly expedite the discovery and optimization of novel active compounds that would be missed using conventional structure-guided drug discovery.

## SUPPLEMENTAL MATERIAL

Supplemental material aac.00909-24-s0001.pdf Tables S1 and S2, Fig. S1 to S6, and Methods S1 to S5.

ASM does not own the copyrights to Supplemental Material that may be linked to, or accessed through, an article. The authors have granted ASM a non-exclusive, world-wide license to publish the Supplemental Material files. Please contact the corresponding author directly for reuse.
